# An-jun-ning, a traditional herbal formula, attenuates spontaneous withdrawal symptoms via modulation of the dopamine system in morphine-dependent rats

**DOI:** 10.1186/1472-6882-14-308

**Published:** 2014-08-19

**Authors:** Jin-Long Gao, Shao-Ang Tu, Jia Liu, Jin-Ming Zhang, Yiyun Huang, Mei Han, Jian-Hui Liang

**Affiliations:** Key Laboratory of Radiopharmaceuticals, Ministry of Education, Department of Chemistry, Beijing Normal University, Beijing, 100875 China; Chinese PLA General Hospital, Beijing, China; Yale PET Center, Department of Diagnostic Radiology, Yale University School of Medicine, New Haven, CT USA; Department of Neuropharmacology, National Institute on Drug Dependence, Peking University, Beijing, 100191 China

**Keywords:** Morphine dependence, Dopamine transporter, Dopamine D_2_receptor, Tyrosine hydroxylase, An-jun-ning

## Abstract

**Background:**

The dopamine system, which is involved in drug dependence, can be damaged by opioid abuse. However, current clinical medicines cannot reverse these damages in the brain, which are believed to be a key reason for the high relapse rate after abstinence treatment. This study aimed to investigate the effects of An-jun-ning (AJN), a commercial traditional Chinese medicine formula used for the treatment of opioid addiction, on the dopamine system in morphine-dependent rats and to explore the possible mechanism underlying its therapeutic effects.

**Methods:**

The morphine dependence model was obtained through injections of morphine at increasing doses for 8 days. The AJN pre-treatment group was administered AJN 30 min before each morphine administration, and the AJN post-treatment groups were treated with AJN for 10 days after withdrawal. Spontaneous withdrawal symptoms (wet dog shakes, and episodes of writhing) were observed after withdrawal. Autoradiography study and/or immunohistochemical staining were used to examine the levels of dopamine transporter (DAT), dopamine D_2_ receptor (D_2_R) and tyrosine hydroxylase (TH).

**Results:**

(1) Pre-treatment with AJN attenuates wet dog shakes and episodes of writhing to approximately 50% or less of those observed in the morphine group (p < 0.01). (2) AJN post-treatment dose-dependently reduced the number of wet dog shakes (p < 0.01), and the episodes of writhing (p < 0.01). (3) Pre-treatment with AJN effectively interdicted the morphine-induced decreases in the levels of DAT, D_2_R, and TH in the striatum (p < 0.01) such that they remained at nearly normal levels. (4) Post-treatment with AJN restored DAT and D_2_R to the normal levels (p < 0.01) and the level of TH to 87% of normal in the striatum.

**Conclusions:**

AJN can effectively alleviate opioid withdrawal symptoms and preserve or restore the DAT, D_2_R, and TH levels in the striatum. The mechanism underlying the effect of AJN on withdrawal symptoms may be related to the modulation of the dopamine system by AJN. These results suggest that AJN may help to prevent relapse in opioid dependence treatment.

## Background

Opioid abuse and dependence is a chronic brain disorder and imposes severe medical and economic burdens on individuals and society. The United Nations Office on Drug and Crime estimates that 12 to 21 million people abuse opiates worldwide [1]. It has been reported that approximately 50% of patients experienced relapse behavior within a few days after completing treatment in a hospital [2]. Another follow-up investigation found that even when presenting to buprenorphine treatment, youth with opioid use within the past 30 days were less likely to avoid relapse at week 12 of treatment [3]. At present, there are some first-line medicines for the treatment of opioid addiction that effectively alleviate the opioid withdrawal symptoms. These include methadone, buprenorphine, naloxone, naltrexone, lofexidine and clonidine. But most of these drugs have undesirable side effects, such as abuse potential and high relapse rate and cannot effectively reverse the adaptive neurobiological changes. Therefore, more effective and safer approaches for the treatment of opioid addiction are urgently needed.

Traditional Chinese medicines (TCMs) have long been used to treat opioid addiction, and many prescriptions were proved to have therapeutic efficacy [4, 5]. An-jun-ning (AJN) is one of the TCMs approved by the China Food and Drug Administration for treatment of opioid addiction. Clinical studies have demonstrated that AJN is safe and well-tolerated by patients [6], and that it effectively alleviates the protracted withdrawal symptoms in heroin users [7, 8]. Further, AJN has been reported to attenuate the impairments in tyrosine hydroxylase (TH) and glial fibrillary acidic protein in the ventral tegmental area, thus potentially implicating the dopamine (DA) system in its therapeutic efficacy [9]. However, the exact mechanism underlying the effects of AJN has yet to be fully elucidated.

The DA system is believed to play an important role in addictive behaviors, including opioid addiction [10–12]. Long-term opioid abuse results in adaptive neurobiological changes in the brain, particularly in the DA system. For example, opioid abuse has been shown to be associated with decreased densities of dopamine transporters (DAT) and dopamine D_2_ receptors (D_2_R) in both animals and humans, and reduced TH activity in rats [13–17]. Current treatments for opioid addiction cannot reverse these neurobiological alterations, and can even cause exacerbation in some cases, resulting in high relapse rate after detoxification [18]. These neurobiological changes are therefore believed to be the key reason for the difficulties associated with abstinence treatment.

In this study, we examined the effects of AJN on the protracted withdrawal symptoms in morphine-dependent rats and sought to determine whether the morphine-induced decreases in DAT, D_2_R and TH can be interdicted (pre-treatment) or alleviated (post-treatment) by AJN. In another word, we attempted to investigate the hypothesis that modulation of the dopamine system by AJN is a possible mechanism of its therapeutic action.

## Methods

### Animals

Male Wistar rats weighing 180–220 g (Academy of Military Medical Science, Beijing, China) at the beginning of the experiment were used. The rats were housed in groups of five in a room with constant temperature (25°C) and humidity (70%) and a 12 h light/12 h dark cycle (08:00–20:00 light on), with free access to food and water. Animals were maintained according to the international guidelines for the care and use of laboratory animals, and all experimental procedures involving animals were approved by the Ethics Committee of Beijing Normal University (BNU/EC/01/2011).

### Drugs and reagents

AJN was provided by Taier Company (Hunan, China). Morphine hydrochloride was purchased from Qinghai Pharmaceutical Co. (China). (*S*)- *N*-((1-ethyl-2-pyrrolidinyl) methyl)-2-hydroxy-6-methoxy-3-(trimethylstannyl) benzamide (TBZM), and (1*R*,2*S*,3*S*,5*S*)-methyl 8-methyl-3-(4-(trimethylstannyl) phenyl)-8-azabicyclo [3.2.1] octane-2-carboxylate (trimethylstannyl-β-CT, or TMS-β-CT) were purchased from Huayi Isotope Co. (Toronto, ON, Canada). Na^125^I (specific activity > 2200 Ci/mmol) was purchased from Perkin Elmer (Boston, MA, USA). [^125^I]-IBZM and [^125^I]-β-CIT were prepared as described by Kung et al., and Toyama et al. [19, 20].

### Morphine dependence model and AJN treatment

The rats were divided into six groups: control group, morphine group, AJN pre-treatment group (0.555 g/kg), and three AJN post-treatment groups: low dose (AJN-L, 0.185 g/kg), medium dose (AJN-M, 0.555 g/kg), and high dose (AJN-H, 1.851 g/kg). The rats in the morphine, AJN pre-treatment and AJN post-treatment groups were administered morphine via intraperitoneal injection twice daily (09:00 and 15:00) for eight days in a volume of 1 mL/kg body weight, with a gradually increasing dose (10, 10, 15, 15, 15, 20, 20, and 20 mg/kg per injection on each day) [21, 22]. The control animals received 0.9% saline in the same volume. AJN was dissolved in deionized water and intragastrically administered to the rats in the AJN pre-treatment group at a dose of 0.555 g/kg (1 mL/kg of body weight) 30 min before each morphine injection. After morphine administrations, the rats in the AJN post-treatment groups were administered AJN intragastrically once daily at a dose of 0.185, 0.555 or 1.851 g/kg (1 mL/kg of body weight) for 10 days, whereas the other groups received the same volume of vehicle (saline). The AJN dosages were converted from those used clinically.

### Behavior observation

At 10:00 a.m. on days one, five, and ten after the withdrawal of morphine, the animals were placed individually into Plexiglas cages and observed for signs of spontaneous withdrawal. Following a 5 min acclimation in the cages, the number of wet dog shakes and writhing episodes were monitored during a 30 min period [23]. Three observers blind to the groups completed the observation and score independently. Scores were averaged for each behavior test.

### Tissue preparation

After the final behavioral observation, all the rats were sacrificed by decapitation. Rat brains were rapidly removed and stored at -80°C until use. The brains were then cut into 18 μm coronal slices with a cryostat (CM1900, Leica, Germany) at -20°C.

### Immunohistochemical staining

Immunohistochemical staining to determine levels of DAT and D_2_R proteins and TH activity was conducted with striatal slices. To prepare for staining, the slices were prewashed in PBS (0.01 M, pH 7.4) three times for 5 min each, then sequentially treated with 0.2% Triton X-100 in PBS for 5 min and with 0.3% H_2_O_2_ in PBS for 10 min, and washed in PBS three times for 5 min each, all at room temperature. Slices were initially incubated with 10% normal goat serum for 10 min (or normal donkey serum for TH measurement), then incubated for 20 h at 4°C with the primary antibodies (D_2_R antibody AB5084P, Millipore, CA, USA, 1:200 dilution; DAT antibody MAB369, Millipore, CA, USA, 1:100 dilution; TH antibody T1299, Sigma, CA, USA, 1:10,000 dilution), washed three times in PBS, and incubated for 60 min at 37°C with the secondary antibodies (anti-rabbit antibody PV-6001, ZSGB-BIO, CA, USA; anti-rat antibody ZB-2307, ZSGB-BIO, CA, USA; anti-mouse antibody PK4002, Vector, CA, USA). Slices were then washed five times for 3 min each in PBS, and incubated in 100 μL of 3,3′-diaminobenzidine tertrahydrochloride (DAB) for 3 min. The slices immunostained for TH were incubated in the ABC (VECTASTAIN ABC kit, ZSGB-BIO, Beijing, China) reagent for 30 min at 37°C and washed five times for 3 min each in PBS prior to the DAB treatment. Final wash of the slices was done in distilled water.

### Autoradiography experiments

The striatal slices were washed for 20 min at room temperature in 50 mM Tris buffer (pH 7.4, containing 120 mM NaCl, 5 mM KCl, 2 mM CaCl_2_, and 1 mM MgCl_2_) for D_2_R autoradiography, and in 50 mM Tris buffer (pH 7.4, containing 120 mM NaCl and 5 mM KCl) for DAT autoradiography. The slices were then incubated for 60 min in the same buffer with 50 pM [^125^I]-IBZM for D_2_R labeling or 50 pM [^125^I]-β-CIT in the presence of 1 mM fluoxetine (serotonin antagonist, Sigma-Aldrich Co., USA) for DAT labeling. The nonspecific binding was determined in the adjacent slices in the presence of 10 μM sulpiride (D_2_R antagonist, Sigma-Aldrich Co., USA) for D_2_R or in the presence of 100 mM nomifensine (DAT antagonist, Sigma-Aldrich Co., USA) and 100 mM fluoxetine for DAT. After incubation, the slices were washed five times for 1 min each in ice-cold 50 mM Tris buffer (pH 7.4), rapidly dipped in deionized water and dried under a stream of cold, dry air.

The labeled slices were mounted and exposed to a super-sensitive phosphor screen (PerkinElmer, USA) at room temperature for 8 h. Densitometry determinations were performed using a Cyclone Plus phosphor imager (PerkinElmer, USA) and analyzed using the Opti-Quant software (PerkinElmer, USA). The specific binding detected in each structure was quantified by subtracting the non-specific binding image from the total binding image. The results are shown as the ratio of specific binding relative to the control group.

### Statistical analysis

All of the statistical analyses were conducted using SPSS software (version 20.0) with a type I error rate of α = 0.05 (two-tailed). Data are expressed as the mean ± SD. The significance of the changes in the behavioral assays was determined using the Kruskal-Wallis test. If the difference was found to be significant using this test, the Mann–Whitney U-test was used to compare the control and experimental groups. To compare the values obtained from two groups, Student’s t-test was performed.

## Results

### Effects of AJN on wet dog shakes and episodes of writhing

We chose to use the spontaneous withdrawal model in this study because its course more closely follows that observed in the clinic than other withdrawal models, such as the naloxone-precipitated withdrawal model. Spontaneous withdrawal from chronic morphine treatment only induced mild signs after withdrawal. Abstinence was commonly evidenced by wet dog shakes and episodes of writhing.

**Figure 1:**
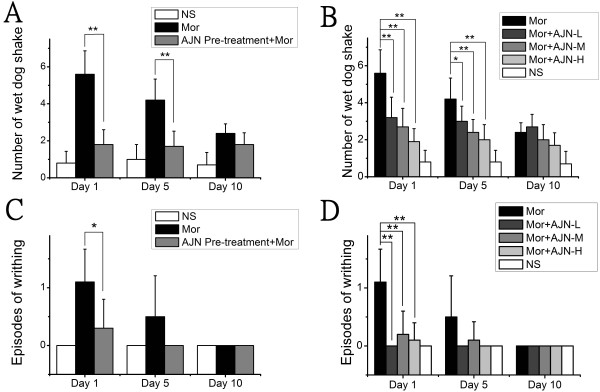
**Effects of AJN on wet dog shakes and episodes of writhing after spontaneous withdrawal. A**: AJN pre-treatment effectively holds wet dog shakes at a low level after 1, 5, 10 days of spontaneous withdrawal. **B**: AJN post-treatment alleviates wet dog shakes in a dose-dependent manner. **C**: AJN pre-treatment inhibits episodes of writhing after spontaneous withdrawal. **D**: AJN post-treatment attenuates episodes of writhing. NS: control group; Mor: morphine group. Data are expressed as the means ± SD (*p < 0.05, **p < 0.01 vs. morphine group; n = 10; Kruskal-Wallis test and Mann–Whitney U-test).

As shown in Figure 1 (A and B), one day after withdrawal, the rats treated with morphine experienced many wet dog shakes. The number of wet dog shakes in the morphine group was 5.6 ± 1.3, which is significantly higher than that observed in the control group (0.8 ± 0.6). AJN pre-treatment significantly reduced the number of wet dog shakes, to 1.8 ± 0.8. Additionally, AJN post-treatment alleviated wet dog shakes in a dose-dependent manner: animals treated with the low dose (AJN-L, 0.185 g/kg), medium dose (AJN-M, 0.555 g/kg) and high dose (AJN-H, 1.851 g/kg) AJN experienced 3.2 ± 1.1, 2.7 ± 1.0, and 1.9 ± 0.7 of wet dog shakes, respectively (Figure 1A and B). Five days after withdrawal, the number of wet dog shakes in the morphine group was 4.2 ± 1.1, whereas animals in the AJN pre-treatment and post-treatment groups all exhibited lower numbers of wet dog shakes. Ten days later, at the end of the experiment, the rats in the morphine group still experienced a higher number of wet dog shakes than those in the control group, while those in the AJN-H post-treatment group experienced significantly lower number.

Episodes of writhing were observed after morphine withdrawal. The rats in the morphine group experienced 1.1 ± 0.6 episodes of writhing, whereas no rats in the control group showed this symptom. AJN pre-treatment effectively reduced the episodes of writhing to a rate of 0.3 ± 0.5 (p < 0.05, compared with the morphine group) (Figure 1C). Rats post-treated with AJN also experienced fewer episodes of writhing than those in the morphine group: AJN-L, AJN-M, and AJN-H treatment resulted in 0 ± 0, 0.2 ± 0.4 and 0.1 ± 0.3 episodes of writhing, respectively (Figure 1D). Five days after withdrawal, very few rats showed an episode of writhing (Figure 1C and D).

### Effect of AJN on DAT, D_2_R and TH expression in the striatum

#### AJN effect on DAT

**Figure 2:**
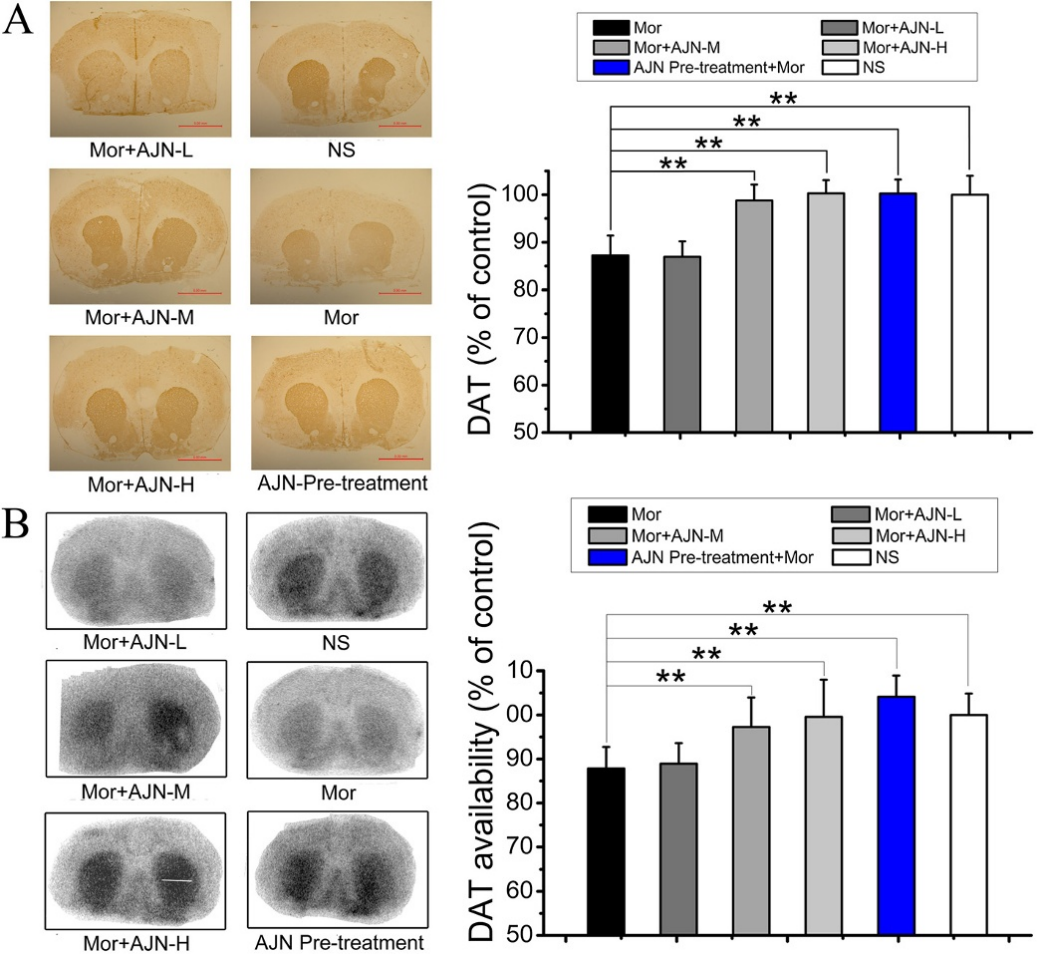
**Effect of AJN pre-treatment on DAT by immunohistochemical staining and autoradiography. A**: Representative staining pictures and effect of AJN on DAT by immunohistochemical staining. **B**: Representative autoradiograms and effect of AJN on DAT by autoradiography with [^125^I]-β-CIT. Results of the two methods are accordant with each other. AJN pre-treatment completely blocks morphine-induced DAT expression decrease, and AJN post-treatment effectively restores DAT expression. NS: control group; Mor: morphine group. Data are expressed as the means ± SD (*p < 0.05, **p < 0.01, vs. morphine group; n = 5; Student’s t-test).

Immunohistochemistry examination revealed that morphine administration in rats decreased the level of DAT to 87.2 ± 4.1% of that observed in the control group (p < 0.01, compared with the control group, Figure 2A). AJN pre-treatment completely inhibited this decrease and DAT expression was maintained 100.2 ± 2.9% of the level observed in the control group, which was significantly higher than that in the morphine group. Additionally, groups post-treated with AJN-L, AJN-M, and AJN-H displayed DAT expression at 86.9 ± 3.2%, 98.8 ± 3.3% and 100.3 ± 2.8% of the level observed in the control group, respectively, indicating that post-treatment with AJN-M and AJN-H normalized DAT expression in morphine dependent animals.

The immunohistochemistry results were confirmed by autoradiographic studies (morphine group: 87.8 ± 4.8% of the control level; AJN pre-treatment: 104.1 ± 4.7% of the control level; AJN-L: 88.9 ± 4.6% of the control level; AJN-M: 97.2 ± 6.7% of the control level; AJN-H: 99.5 ± 8.4% of the control level) (Figure 2B).

#### AJN effect on D_2_R

**Figure 3:**
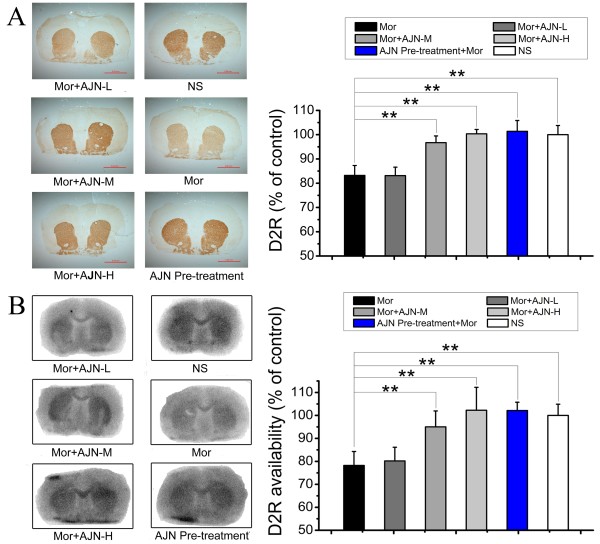
**Effect of AJN on D**_**2**_ **R by immunohistochemical staining and autoradiography. A**: Representative staining pictures and effect of AJN on D_2_R by immunohistochemical staining. **B**: Representative autoradiograms and effect of AJN on D_2_R by autoradiography with [^125^I]-IBZM. Morphine administration reduces D_2_R expression by about 20%. AJN pre-treatment completely inhibits the morphine-induced D_2_R decrease. And AJN post-treatment alleviate the decrease in D_2_R expression in a dose-dependent manner. NS: control group; Mor: morphine group. Data are expressed as the means ± SD (*p < 0.05, **p < 0.01, vs. morphine group; n = 5; Student’s t-test).

Immunohistochemical staining revealed that chronic morphine administration reduced D_2_R expression in the striatum to 83.2 ± 4.0% of the level in the control group (p < 0.01, compared with the control group, Figure 3A). AJN pre-treatment completely countered against this decrease and maintained DAT expression at 101.3 ± 4.4% of the level observed in the control, which is significantly different from the level observed in the morphine group. Additionally, post-treatment with AJN-L, AJN-M, and AJN-H resulted in D_2_R expression at 83.1 ± 3.5%, 96.7 ± 2.7% and 100.3 ± 1.7% of the level observed in the control group, indicating that both AJN-M and AJN-H post-treatments recovered D_2_R expression to the normal level. These findings by immunohistochemical staining were confirmed by autoradiography (morphine group: 78.2 ± 6.0% of the control level; AJN pre-treatment: 102.1 ± 3.6% of the control level; AJN-L: 80.1 ± 5.9% of the control level; AJN-M: 95.0 ± 6.9% of the control level; AJN-H: 102.2 ± 9.9% of the control level) (Figure 3B).

#### AJN effect on TH reactivity

**Figure 4:**
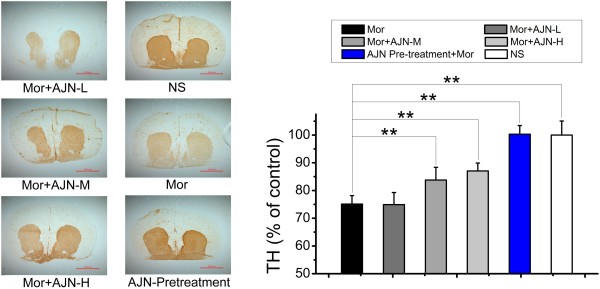
**Effect of AJN on TH density by immunohistochemical staining.** Representative staining pictures and effect of AJN on TH density by immunohistochemical staining. TH density in the striatum is decreased in the morphine group. Both AJN pre- and post-treatment effectively attenuates this decrease. NS: control group; Mor: morphine group. Data are expressed as the means ± SD (*p < 0.05, **p < 0.01, vs. morphine group; n = 5; Student’s t-test).

In the morphine group, TH reactivity in the striatum was reduced to 75.1 ± 3.0% of the level in the control group (p < 0.01, compared with the control group, Figure 4). AJN pre-treatment completely inhibited this decrease and maintained TH expression at 101.3 ± 4.4% of the level in the control group, which is significantly different from that observed in the morphine group. Additionally, AJN post-treatment alleviated the decrease in TH expression in a dose-dependent manner: TH expression levels in the rats post-treated with AJN-L, AJN-M, and AJN-H were 74.9 ± 4.3%, 83.7 ± 4.6%, and 87.0 ± 2.8%, respectively, of the control level.

## Discussion

This study provides the first preclinical investigation on the modulation of the dopamine system as a mechanism underlying the therapeutic action of the traditional Chinese medicine formula An-jun-ning in alleviating spontaneous withdrawal symptoms in opioid dependence. Results from the present study demonstrated that AJN effectively alleviated the morphine withdrawal symptoms. Immunohistochemical and autoradiographic studies indicated that pre-treatment with AJN inhibited the morphine-induced decreases in DAT, D_2_R, and TH expression in the striatum, suggesting that a mechanism of action for AJN might be related to its modulation of the dopamine system. Additionally, post-treatment with medium or high dose of AJN normalized DAT and D_2_R expression in morphine dependent rats, which confirms that AJN can effectively act on the dopamine system. Taken together, these results provide support for the hypothesis that the mechanisms by which AJN mitigates morphine withdrawal symptoms involve modulation of the DA system.

The restoration of DA function is believed to be beneficial in the treatment of opioid addiction and can result in reduced relapse rate. There is considerable evidence for the dysregulation of DA system following repeated drug intake, and the changes observed persist throughout the early phases of abstinence. Chronic administration of morphine or heroin to rodents has been shown to cause decreases in striatal concentrations of synaptic DA, TH, DAT, and D_2_R [15, 24–27]. Decreased levels of DA, DAT and D_2_R have also been found in patients addicted to heroin [16–18]. Collectively, these findings are consistent with the hypothesis that an effective treatment for opioid addiction may be achieved if the neurologic impairments are alleviated and DA function is restored [28]. In this study, we examined the effects of pre- and post-treatment with AJN on striatal DAT, D_2_R, and TH levels in a rat model of morphine-dependence. Our results demonstrated that 1) morphine dependence induced decreases in DAT, D_2_R and TH levels in the striatum; 2) these decreases were effectively inhibited by pre-treatment with AJN; and 3) post-treatment with AJN acted against these decreases, with medium and high dose AJN post-treatment restoring DAT, D_2_R in the morphine model rats to the normal levels. Taken together, results from our present study support the hypothesis that the dopamine system plays an important role in the therapeutic mechanism of AJN and thus provide a novel strategy for the treatment of opioid addiction.

Based on previous studies of *Rhizoma Corydalis*, which is one of the major ingredients in AJN, we postulate that the effect of AJN on the dopamine system might be partly related to the compounds found in *Rhizoma Corydalis* [29]. Previous studies showed that *l*-tetrahydropalmatine (*l*-THP), the primary active component in *Rhizoma Corydalis*, attenuated the conditioned place preference in morphine-dependent rats, and acted on the D_2_R in the striatum to enhance endogenous opioid peptide function, and hence accelerated the functional recovery of the dopamine system [30–32]. These studies provided support for the hypothesis that *l*-THP might play an important role in the therapeutic effects of AJN on the dopamine system. On the other hand, since AJN is a traditional Chinese medicine formula with multiple other components in addition to *Rhizoma Corydalis* (Table 1), it is reasonable to contemplate that, besides *Rhizoma Corydalis* and *l*-THP, other components and ingredients may also contribute to its efficacy in alleviating morphine withdrawal symptoms, and its protective/restorative effects on dopamine function. Much work remains to be done to fully elucidate the pharmacological targets involved in the therapeutic action of AJN.

**Table 1:** **Composition of AJN**

Scientific name	Amount (%)
*Rhizoma Corydalis*	39.78
*Radix Paeoniae Alba*	15.75
*Glycyrrhiza uralensis Fisch*	2.39
*Syringa Linn*	2.39
*Houttuynia cordata Thunb*	11.93
*Terminalia chebula Retz*	11.93
*Radix Cynanchi Paniculati*	3.98
*Alpinia oxyphylla*	3.98
*Arisaema cum bile*	7.87
Total amount	100.00

Nonetheless, results from the present study provide the first evidence that AJN may exert its therapeutic action for the treatment of opioid dependence through modulation of the dopamine system, as we demonstrated that AJN not only protects the DA system from the deleterious effects of morphine (through pretreatment studies), but also counteracts against these effects (via post-treatment studies), both of which involve the maintenance of DAT, D_2_R and TH at normal levels.

Availability of DAT and D_2_R can be detected clinically through molecular imaging methods, such as positron emission tomography (PET) and single photon emission computed tomography (SPECT) [14, 16]. In the current study we examined DAT and D_2_R levels via autoradiography, which is based on the same principles as PET and SPECT imaging. Results from autoradiography studies are corroborated by and consistent with those obtained through immunohistochemical staining, which is an accepted protein detection technique. Together, these results provide strong preclinical evidence for the ability of AJN to simultaneously counter against the morphine-induced decreases in the levels of DAT, D_2_R, and TH in the striatum and to offer relief from morphine withdrawal symptoms. Hence, our study offers new insights into the mechanism of AJN therapeutic action and implies the modulation of dopamine system as an effective avenue for the treatment of opioid addiction. Future clinical studies using PET or SPECT imaging will serve to verify these findings in patients and advance our understanding and development of effective therapeutic interventions in opioid dependence.

## Conclusions

In this report we demonstrate that the traditional Chinese medicine formula An-jun-ning effectively alleviates the spontaneous morphine withdrawal symptoms. Further, AJN is shown to normalize DAT, D_2_R and TH levels in the striatum of morphine dependent animals. Hence, this study offers the first demonstration of AJN’s effects on the dopamine system, and thus provides insights into the clinical benefits of AJN treatment for opioid addiction. Further studies are warranted to examine AJN therapeutic effects on the dopamine system in the clinic.

## Abbreviations

AJN: An-jun-ning (a traditional Chinese medicine)
DA: Dopamine
DAT: Dopamine transporter
D_2_R: Dopamine D_2_ receptor
TH: Tyrosine hydroxylase
TCM: Traditional Chinese medicine
*l*-THP: *l*-tetrahydropalmatine
PET: Positron emission tomography
SPECT: Single photon emission computed tomography.
